# Late infantile and adult‐onset metachromatic leukodystrophy due to novel missense variants in the *PSAP* gene: Case report from India

**DOI:** 10.1002/jmd2.12374

**Published:** 2023-06-05

**Authors:** Jayesh Sheth, Aadhira Nair, Riddhi Bhavsar, Heli Shah, Naresh Tayade, C. Ratna Prabha, Frenny Sheth, Harsh Sheth

**Affiliations:** ^1^ FRIGE's Institute of Human Genetics, FRIGE House Ahmedabad India; ^2^ Smt. NHL Municipal Medical College Ahmedabad India; ^3^ Department of Pediatrics Dr. Panjabrao Deshmukh Memorial Medical College Amravati India; ^4^ Department of Biochemistry, Faculty of Science The M. S. University of Baroda Vadodara India

**Keywords:** case report, gait, MLD, prosaposin, smMIP‐NGS, tremors

## Abstract

Metachromatic leukodystrophy (MLD) due to Sap‐B deficiency is a rare autosomal recessive disorder caused due to biallelic variants in the *PSAP* gene. The *PSAP* gene encodes a precursor protein prosaposin, which is subsequently cleaved to form four active glycoproteins: Sap‐A, Sap‐B, Sap‐C, and Sap‐D. In case of deficiency of the sphingolipid activator protein Sap‐B, there is a gradual accumulation of cerebroside‐3‐sulfate in the myelin of the nervous system resulting in progressive demyelination. Only 12 variants have been reported in the *PSAP* gene causing Sap‐B deficiency to date. Here, we report two cases of MLD due to Sap‐B deficiency (late‐infantile and adult‐onset form) harboring two novel missense variants c.688T > G and c.593G > A in the *PSAP* gene respectively. This study reports the third case of adult‐onset MLD due to Sap‐B deficiency in the world. The proband, a 3‐year‐old male child presented with complaints of hypotonia, lower limb tremors and global developmental delay. His MRI showed hyperintense signals in the bilateral cerebellar white matter. Overall, the findings were suggestive of metachromatic leukodystrophy. The second case was a 19‐year‐old male child with clinical features of regression of speech, gait ataxia and bilateral tremors referred to our clinic. MRI data suggested metachromatic leukodystrophy. Normal enzyme activity of arylsulfatase‐A led to a suspicion of saposin B deficiency. For both cases, targeted sequencing was performed. This identified homozygous variant c.688T > G (p.Cys230Gly) and c.593G > A (p.Cys198Tyr) in exon 6 of the *PSAP* gene, respectively.

**Synopsis**

We describe two cases of MLD due to novel pathogenic variants in the *PSAP* gene from India. Overall, we conclude that MLD due to Sap‐B deficiency should be considered as a differential diagnosis in adult patients presenting with speech regression, gait, and bilateral tremors.

## 1 INTRODUCTION

Metachromatic leukodystrophy (MLD) is an autosomal recessive neurodegenerative disorder. It is characterized by psychomotor regression, gait disturbances, ataxia, spastic paraparesis, and visual disturbances.^1^ The breakdown of the substrate is catalyzed by the lysosomal enzyme Arylsulfatase‐A (E.C.3.1.6.8) in presence of a sphingolipid activator protein, saposin‐B (Sap‐B).^1^, ^2^ The gradual accumulation of cerebroside‐3‐sulfate particularly in the myelin of the nervous system results in progressive demyelination and dysfunction of the central nervous system and the peripheral nervous system respectively. Any defect in the genes *ARSA* or *PSAP* which encode the Arylsulfatse‐A enzyme and Sap‐B protein respectively can result in MLD. The majority of the MLD cases are due to pathogenic variants in the *ARSA* gene causing MLD due to ARSA deficiency (OMIM#250100). A small group of 30 MLD patients due to deficiency of the activator protein Sap‐B has been reported (OMIM#249900). The key distinguishing feature between these two groups is that arylsulfatase‐A enzyme levels in Sap‐B patients are within the normal range as opposed to that in ARSA deficiency patients. However, in both conditions, there is an abnormal excretion of urine sulfatide.^3^

The *PSAP* gene (MIM#176801) located on chromosome 10 consisting of 14 coding exons encodes a 524 amino acid precursor protein prosaposin (pSap) which is subsequently cleaved to form four active glycoproteins (Sap‐A, Sap‐B, Sap‐C, and Sap‐D). Mutations in the *PSAP* gene can lead to a deficiency of the entire pSap protein or individual saposins.^3^ MLD is due to a defect in Sap‐B whereas Sap‐A and Sap‐C defects cause atypical Krabbe disease and Gaucher disease respectively.^4^, ^5^, ^6^ Sap‐D deficiency has been reported only in mice and it resembles Farber disease.^7^

To date, a total of 12 variants in the PSAP gene have been reported in 30 MLD patients worldwide.^1^ In India, two cases of MLD due to Sap‐B deficiency have been reported.^8^, ^9^ Both were late infantile forms and showed the presence of the variant c.679_681delAAG in exon 6 of the *PSAP* gene. The severity of the condition is governed by the type of variant.^1^ Clinically, the most severe prosaposin deficit is due to two *PSAP* null alleles. Of all the Sap‐B deficient MLD patients reported, the majority of them presented with the late infantile form (17 patients) followed by 6 cases of juvenile‐onset and two cases of the adult‐onset form.^10^, ^11^

The three most common PSAP‐MLD alleles reported are c.645C > A, c.722G > C, and c.577‐1G > T, and these account for about 67% of the total PSAP‐MLD alleles.^1^ Overall, 4 missense variants have been reported in the *PSAP* gene and they have been associated with both late‐infantile as well as juvenile forms of MLD due to Sap‐B deficiency. Here, we report two cases: a late‐infantile form and an adult‐onset form of MLD due to novel missense variants in the *PSAP* gene. To the best of our knowledge, this study describes the third report in the world and the first from India, of an adult‐onset MLD phenotype resulting from Sap‐B deficiency.

## 2 CASE REPORT

Case 1 is a 3‐year‐old male child born to consanguineous parents. On presentation to the clinician, global developmental delay, hypotonia, lower limb tremors and poor feeding was noted. There was a history of recurrent fever, cold, and cough since 1 year of age. The parents complained that the child had become more irritable in the last 6 months. He was able to walk with support and speak monosyllables at the age of 2 years. Thereafter, parents noticed regression of learned skills. Brain MRI studies of the proband showed moderate confluent FLAIR/T2 hyperintense signal in the periventricular and deep white matter of bilateral frontal, parietal, and occipital lobes. In addition, FLAIR/T2 hyperintense signal was noted in corpus callosum involving genu and splenium. A mild FLAIR hyperintense signal was also noted in the bilateral deep cerebellar white matter. Overall, the MRI findings were suggestive of metachromatic leukodystrophy (MLD).

For molecular confirmation, genomic DNA was extracted from peripheral blood using salting out technique and was subjected to a targeted exome sequencing study as mentioned in the online supplementary file. Variant filtration and prioritization analysis revealed a homozygous variant c.688T > G (p.Cys230Gly) in exon 6 of the *PSAP* gene (NM_002778.4). This variant has not been reported in the 1000 genomes^12^ and gnomAD databases.^13^ The in‐silico prediction of the variant is damaging by SIFT, PolyPhen‐2, PROVEAN, and MutationTaster. The variant was classified as likely pathogenic as per the ACMG‐AMP guidelines and ClinGen framework with the following criteria—PP3 (strong) and PM2 (moderate).^14^, ^15^ This established the diagnosis of MLD due to Sap‐B deficiency in the proband. As the child's sample was not available for Sanger confirmation study, we performed parental segregation analysis. We found both parents to be heterozygous (carrier) for the variant c.688T > G in exon 6 of the *PSAP* gene (Figure S1).

Case 2 is a 19‐year‐old male child born to a phenotypically healthy and an endogamous couple from Rajasthan, presented with gait ataxia, difficulty in walking and nystagmus. His psychomotor development including speech was normal until 14 years of age. At the age of 13 years, his parents noticed him having difficulty in speech. Following this, he started having trouble in walking. He also had a history of febrile illness. His MRI brain revealed confluent and symmetrical areas of T2W/FLAIR hyperintensities in the periventricular and deep white matter of bilateral cerebral hemispheres, centrum semiovale and corpus callosum. Mild thinning of the corpus callosum was also seen. No other abnormality was noted in the cerebral parenchyma, cerebellum, brainstem, and pituitary gland. The MRI results led to an initial suspicion of a case of MLD.

The proband was referred to our Centre at the age of 19 years. In addition to difficulty in walking and speech, he also had a history of tremors in both hands. In order to rule out MLD, enzyme levels of arylsulfatase‐A activity were assessed from leukocytes. Briefly, the arylsulfatase‐A assay was carried out using p‐nitrocatechol as the substrate. The assay mixture was incubated at 0°C for a period of 16 h followed by the addition of 1 N NaOH to stop the reaction. The extent of activity was then measured spectrophotometrically.^16^ He showed a normal enzyme activity of 2.9 nmol/h/mg protein (Normal range: 0.6–4.99). Thus, MLD was ruled out; however, considering the clinical indications, a plausible differential diagnosis of saposin B deficiency was suggested. We then subjected the DNA of the proband to a targeted gene panel study based on the single molecule molecular inversion probes (smMIPs) for 23 genes associated with common lysosomal storage disorders in India.^17^, ^18^, ^19^ A detailed methodology is mentioned in the online supplementary file. This revealed the presence of a homozygous novel missense variant c.593G > A (p.Cys198Tyr) in exon 6 of the PSAP (NM_002778.4) gene (Figure S2). This variant has not been reported in the 1000 genomes^12^ and gnomAD databases.^13^ The in‐silico prediction of the variant is damaging by MutationTaster, PROVEAN, LRT, DANN, and SIFT. The variant was classified as likely pathogenic as per the ACMG guidelines and ClinGen framework with the following criteria‐PP3 (strong), PM1 (supporting), PM2 (supporting).^14^, ^15^ This confirmed the diagnosis of MLD due to Sap‐B deficiency in the proband. Sanger sequencing in the proband confirmed the presence of the homozygous variant c.593G > A in the *PSAP* gene (Figure S3). However, the family was lost to follow‐up and hence there is a paucity of MRI images and parental segregation studies.

## 3 DISCUSSION

MLD is a storage disorder characterized by the gradual accumulation of sulfatides that trigger demyelination. MLD caused due to sulfatide activator protein deficiency encoded by the PSAP gene is relatively rare with only 12 causative variants reported globally (Table 1).^1^, ^10^ The clinical presentation of these patients is similar to patients affected with MLD except that the latter show deficiency of ARSA enzyme activity. The present study describes two cases of MLD due to Sap‐B deficiency from India with novel missense variants in the *PSAP* gene. Also, this is the first report from India describing an adult‐onset MLD phenotype because of Sap‐B deficiency.

**TABLE 1:** Overview of the phenotype and genotype of the PSAP‐MLD cases in the literature along with the present case.

Location	Codon change	Protein change	Variant type	Patient	Clinical phenotype	Nerve biopsy/MRI findings	Age at onset	Reference
Intron 5	c.577‐1G > T	p.Asp193_ Ile240del p.Asp193_ Gln199del	Splicing	1	Motor difficulties	Demyelination, metachromatic deposits in macrophages	2 years	Henseler et al. 1996^23^
				2	Gait disturbances	Symmetrical deep white matter abnormalities	12 months	Grossi et al 2008^24^
Intron 5	c.577‐2G > T	p.Asp193_ Ile240del p.Asp193_ Gln199del	Splicing	3	Abnormal movements, hypotonia	Medial artery infarction, retarded myelination	7 months	Kuchar et al. 2009^25^
**Exon 6**	**c.593G > A**	**p.Cys198Tyr**	**Missense**	**4**	**Regression of speech, gait disturbance, bilateral tremors**	**Symmetrical areas of hyper intensities in periventricular and deep white matter region**	**13 years**	**This study**
Exon 6	c.643A > C	p.Asn215His	Missense	5a	Motor deterioration, hypotonia, weakness	Active demyelination and metachromatic deposits in macrophages.	2 years	Wrobe et al. 2000^4^
				5b	Hypotonia	Initial occipital demyelination	2 years	Wrobe et al. 2000^4^
Exon 6	c.645C > A	p.Asn215Lys	Missense	6	Walking difficulties, speech regression, loss of fine motor skills	Diffuse hyper intense signal in periventricular and subcortical white matter on T2 weighted images	2 years	Regis et al. 1999^21^
				7	Gait disturbance, dysarthria, irritability and weakness	Diffuse abnormal T2 prolongation in the deep cerebral white matter, suggestive of leukodystrophy	1.8 years	Deconinck et al. 2008^26^
				8	Gait disturbance	Symmetrical deep WM abnormalities	2.6 years	Grossi et al. 2008^24^
				9	Gait disturbance	Symmetrical deep WM abnormalities	2.3 years	Grossi et al. 2008^24^
				10	Gait disturbance	Symmetrical deep WM and basal ganglia changes	2.4 years	Grossi et al. 2008^24^
				11	Gait disturbance	NA	3.8 years	Cesani et al. 2016^1^
				12	Behavioral changes with aggressiveness	Diffuse leukoencephalopathy	22 years	Fenu et al. 2019^11^
Exon 6	c.650C > T	p.Thr217Ile	Missense	13a	Behavioral abnormalities	NR	4.6 years	Kretz et al. 1990^27^, ^28^
				13b	Generalized seizure	NR	6 years	Kretz et al. 1990^27^, ^28^
Exon 6	c.665T > C	p.Leu222Ser	Missense	12	Behavioral changes with aggressiveness	Diffuse leukoencephalopathy	22 years	Fenu et al. 2019^11^
Exon 6	c.679_681delAAG	p. Lys227del	Deletion	14	Walking disturbance	Diffuse hypersignal intensity changes in T2‐weighted and FLAIR images	2 years	Kolnikova et al. 2019^10^
				15	Acute regression of motor milestones	Periventricular white matter demyelination	3.6 years	Madaan et al. 2019^8^
				16	Progressive neurologic deterioration	T2/fluid‐attenuated inversion recovery signal changes in the periventricular and deep white matter with contrast enhancement of the cranial nerve	9 months	Sankaran et al. 2020^9^
**Exon 6**	**c.688T > G**	**p.Cys230Gly**	**Missense**	**17**	**Hypotonia, regression of milestones, walking difficulty, lower limb tremors**	**Moderate confluent FLAIR/T2 hyperintense signal in periventricular and deep white matter of b/l frontal, parietal and occipital lobes**	**2.5 years**	**This study**
Exon 7	c.722G > C	p.Cys241Ser	Missense	18	‐	‐	7 years	Holtschmidt et al. 1991^29^
				19a	Walking difficulties, dysarthria and spasticity	Symmetrical bilateral WM abnormalities in the cerebral hemispheres	4 years	Al‐Hassnan et al. 2009^20^
				19b	Walking difficulties, dysarthria and spasticity	Symmetrical bilateral WM abnormalities in the cerebral hemispheres	4 years	Al‐Hassnan et al. 2009^20^
				20a	Walking difficulties	Symmetrical bilateral WM abnormalities in the cerebral hemispheres	1.6 years	Al‐Hassnan et al. 2009^20^
				20b	Walking difficulties	Symmetrical bilateral WM abnormalities in the cerebral hemispheres	1.3 years	Al‐Hassnan et al. 2009^20^
				20c	Asymptomatic at 6 months	Normal	‐	Al‐Hassnan et al. 2009^20^
				21a	Walking difficulties and spastic lower limbs	Symmetrical bilateral WM abnormalities in the cerebral hemispheres	3 years	Al‐Hassnan et al. 2009^20^
				21b	Walking difficulties and spastic lower limbs	Symmetrical bilateral WM abnormalities in the cerebral hemispheres	3 years	Al‐Hassnan et al. 2009^20^
				21c	Asymptomatic at 2 years	ND	‐	Al‐Hassnan et al. 2009^20^
				22	Motor regression and speech delay	Symmetrical bilateral white matter abnormalities in the cerebral hemispheres	1.8 years	Al‐Hassnan et al. 2009^20^
Exon 7–8	c.777_778ins24^	p.Met259_ Gln260ins8	Splicing	23	Psychomotor retardation	Moderate cerebral atrophy with diminished density in the periventricular region.	22 years	Hahn et al. 1982^30^, ^31^, ^32^
Exon 8	c.828_829delGA	p.Glu276Aspfs*27	Frameshift	3	Abnormal movements, hypotonia	Medial artery infarction, retarded myelination	7 months	Kuchar et al. 2009^25^
Intron 8	c.909 + 1G > A	p.Gln260_Lys303del	Splicing	24a	Respiratory infection, frequent falls, gait	Diffused and symmetrical areas of high signal intensity on T2‐weighted and fluid‐attenuated inversion recovery (FLAIR) images in the periventricular white matter	2.3 years	Siri et al. 2014^33^
				24b	Upper respiratory infection, mild axial hypotonia	T2‐weighted image shows tenuous white matter hyperintensity in the posterior centrum semiovale bilaterally	5 months	Siri et al. 2014^33^
Exon 11	c.1268delT	p.Leu423Argfs*40	Frameshift	14	Walking disturbance	Diffuse hypersignal intensity changes in T2‐weighted and FLAIR images	2 years	(Kolnikova et al. 2019)^10^

*Note*: The bold values represent case specific genetic findings identified in the two patients presented here and provide a contrast against other known cases.

Abbreviations: NA, not applicable; ND, not done; NR, not reported.

The initial symptoms at onset in most of the Sap‐B deficient patients include difficulty in walking, gait disturbance, speech problems and tremors, which are similar to that seen in both cases. The majority of cases reported to date are of patients with late infantile onset where the symptoms developed after ~2 years of age which was also observed in case 1. In the second case, however, the proband had normal development and started presenting early signs of difficulty in walking only after 14 years of age, which suggested an adult‐onset presentation of the condition. The brain MRI results seen in both of our patients are consistent with that seen in MLD patients. Generally, brain MRI findings include signs of demyelination, diffuse white matter changes, and impairment of basal ganglia.^20^ It is crucial to note that the MRI information of patients can aid in the early diagnosis of MLD. For proband in case 2, activity of the arylsulfatase‐A enzyme was observed within the normal range. This is in concordance with previous observations whereby high excretion levels of urinary sulfatides couple with normal activity of arylsulfatase‐A enzyme is observed.^10^ Testing of urinary sulfatide is therefore important to confirm Saposin‐B deficiency in such cases. However, as the proband was lost to the follow‐up, investigation of urinary sulfatides could not be carried out post genetic test.

To date, different types of mutations including missense, nonsense, splicing, frameshift, deletion and start loss have been reported in the *PSAP* gene. Both our patients showed the presence of novel missense variants in exon 6 of the *PSAP* gene. The variants c.688T > G and c.593G > A identified in the present study have been submitted in the ClinVar database under the submission ID, SUB12964537 and SUB12964504 respectively. In addition to this, three missense variants and a 3‐bp deletion have been previously reported in exon 6 of the *PSAP* gene in patients with Sap‐B deficiency. Out of the missense variants reported, c.645C > A is one of the common PSAP‐MLD alleles. This variant was first reported in an Italian patient and was shown to abolish the N‐glycosylation site of saposin B.^21^ Overall, we find maximum variants reported in exon‐6 of the *PSAP* gene suggesting it to be a hotspot region.

**FIGURE 1:**
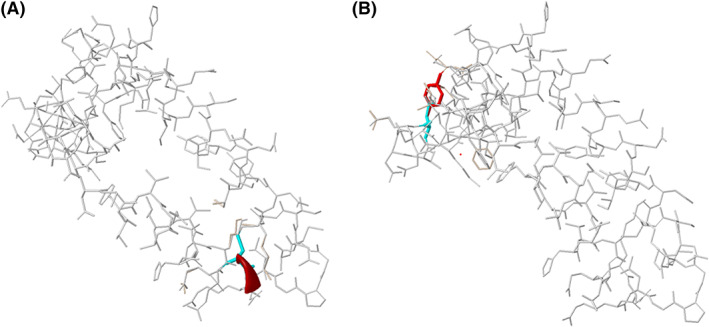
(A) Predicted 3D structure of the PSAP protein due to the variant c.688T > G (Blue: wildtype residue: Cys, Red: mutant residue: Gly) and (B) Predicted 3D structure of the PSAP protein due to the variant c.593G > A (Blue: wildtype residue: Cys, Red: mutant residue: Tyr).

There have been two previous reports of MLD due to Sap‐B deficiency from India. In both cases, the variant c.679_681delAAG in exon 6 of the *PSAP* gene was identified.^8^, ^9^ Both these patients presented with the late‐infantile form. Interestingly, this variant has also been reported in a compound heterozygous state in a late infantile MLD patient.^10^ All patients harboring the c.679_681delAAG variant have shown a similar clinical course. However, it might be essential to analyze the effect of different variant types on the pSap protein in order to relate it to the clinical course of the patient. As both variants identified in this study are novel missense variants, we predicted their effect on the protein by using Missense3D (http://missense3d.bc.ic.ac.uk/~missense3d/, accessed on November 28, 2022). Interestingly, in both cases the wild‐type amino acid that was substituted was cysteine. For case 1, the mutant allele resulted in the substitution of cysteine by glycine. For case 2, there was the substitution of cysteine by tyrosine. The wild‐type cysteine residues at positions 230 and 198 are involved in disulphide bond formation with the cysteine residues at the 47th and 36th positions respectively. Thus, the substitution in both cases disrupts this bond, thereby disturbing the stability of the protein (Figures 1A,B).^22^

Although, the predicted effect of the mutant residue on the protein is similar for both cases, yet there was a difference in the age of onset as well as phenotypic heterogeneity was seen. The former case had an early onset with severe presentation while the latter case had an adult onset. This suggests that there are likely to be other factors that influence the protein function and functional studies can aid in understanding the genotype–phenotype correlation.

In conclusion, the present study adds two novel variants in the existing mutation list for the *PSAP* gene and suggests that exon 6 of the *PSAP* gene is likely to be a hotspot region for mutation. It also highlights the need to consider MLD due to Sap‐B deficiency in the differential diagnosis of patients presenting with speech regression, gait, and bilateral tremors.

## AUTHOR CONTRIBUTIONS

*Conceived and designed experiments*: Jayesh Sheth, Aadhira Nair and Harsh Sheth. *Patient recruitment and clinical analysis*: Jayesh Sheth, Naresh Tayade, Frenny Sheth and Heli Shah. *Enzyme study*: Riddhi Bhavsar. *Sequencing data analysis and interpretation*: Harsh Sheth and Aadhira Nair. *Write first draft of the manuscript*: Aadhira Nair and Jayesh Sheth. *Made critical revisions and approved final version*: Jayesh Sheth, Harsh Sheth, C. Ratna Prabha. All authors reviewed and approved the final manuscript.

## FUNDING INFORMATION

We sincerely acknowledge research funding from the Department of Biotechnology (BT/PR39587/MED/12/851/2020) and the Gujarat State Biotechnology Mission (GSBTM/JDR&D/608/2020/459‐461) for the above work.

## CONFLICT OF INTEREST STATEMENT

Jayesh Sheth, Aadhira Nair, Riddhi Bhavsar, Heli Shah, Naresh Tayade, C. Ratna Prabha, Frenny Sheth and Harsh Sheth declare that they have no conflict of interest.

## ETHICS STATEMENT

The content is solely the responsibility of the authors and does not necessarily represent the official views of the Department of Biotechnology or the Gujarat State Biotechnology Mission. Funding sources had no role in the design or execution of the study, in the interpretation of data or the writing of the study.

## INFORMED CONSENT STATEMENT

All procedures followed were in accordance with the ethical standards of the institutional ethics committee of FRIGE's Institute of Human Genetics (Reg No‐E/13237) and with the Helsinki Declaration of 1975, as revised in 2000. Informed consent was obtained from all patients for being included in the study.

## Supporting information


**Data S1.** Supporting Information

## Data Availability

Data sharing is not applicable to this article as no new data were created or analyzed in this study.

## References

[1] Cesani M , Lorioli L , Grossi S , et al. Mutation update of ARSA and PSAP genes causing metachromatic leukodystrophy. Hum Mutat. 2016;37(1):16‐27. doi:10.1002/humu.22919 26462614

[2] Gieselmann V , Krägeloh‐Mann I . Metachromatic leukodystrophy—an update. Neuropediatrics. 2010;41(1):1‐6. doi:10.1055/s-0030-1253412 20571983

[3] Elleder M , Jerábková M , Befekadu A , et al. Prosaposin deficiency—a rarely diagnosed, rapidly progressing, neonatal neurovisceral lipid storage disease. Report of a further patient. Neuropediatrics. 2005;36(3):171‐180. doi:10.1055/s-2005-865608 15944902

[4] Wrobe D , Henseler M , Huettler S , Pascual Pascual SI , Chabas A , Sandhoff K . A non‐glycosylated and functionally deficient mutant (N215H) of the sphingolipid activator protein B (SAP‐B) in a novel case of metachromatic leukodystrophy (MLD). J Inherit Metab Dis. 2000;23(1):63‐76. doi:10.1023/a:1005603014401 10682309

[5] Spiegel R , Bach G , Sury V , et al. A mutation in the saposin A coding region of the prosaposin gene in an infant presenting as Krabbe disease: first report of saposin A deficiency in humans. Mol Genet Metab. 2005;84(2):160‐166. doi:10.1016/j.ymgme.2004.10.004 15773042

[6] Vaccaro AM , Motta M , Tatti M , et al. Saposin C mutations in Gaucher disease patients resulting in lysosomal lipid accumulation, saposin C deficiency, but normal prosaposin processing and sorting. Hum Mol Genet. 2010;19(15):2987‐2997. doi:10.1093/hmg/ddq204 20484222

[7] Matsuda J , Kido M , Tadano‐Aritomi K , et al. Mutation in saposin D domain of sphingolipid activator protein gene causes urinary system defects and cerebellar Purkinje cell degeneration with accumulation of hydroxy fatty acid‐containing ceramide in mouse. Hum Mol Genet. 2004;13(21):2709‐2723. doi:10.1093/hmg/ddh281 15345707

[8] Madaan P , Jauhari P , Chakrabarty B , Kumar A , Gulati S . Saposin B‐deficient metachromatic leukodystrophy mimicking acute flaccid paralysis. Neuropediatrics. 2019;50(5):318‐321. doi:10.1055/s-0039-1692646 31319425

[9] Parayil Sankaran B , Nagappa M , Chiplunkar S , et al. Leukodystrophies and genetic leukoencephalopathies in children specified by exome sequencing in an expanded gene panel. J Child Neurol. 2020;35(7):433‐441. doi:10.1177/0883073820904294 32180488

[10] Kolnikova M , Jungova P , Skopkova M , et al. Late infantile metachromatic leukodystrophy due to novel pathogenic variants in the PSAP gene. J Mol Neurosci. 2019;67(4):559‐563. doi:10.1007/s12031-019-1259-7 30632081

[11] Fenu S , Castellotti B , Farina L , et al. Saposin B deficiency as a cause of adult‐onset metachromatic leukodystrophy. Neurology. 2019;93(7):310‐312. doi:10.1212/WNL.0000000000007951 31289144

[12] 1000 Genomes Project Consortium , Auton A , Brooks LD , et al. A global reference for human genetic variation. Nature. 2015;526(7571):68‐74. doi:10.1038/nature15393 26432245 PMC4750478

[13] Karczewski KJ , Francioli LC , Tiao G , et al. The mutational constraint spectrum quantified from variation in 141,456 humans. Nature. 2020;581(7809):434‐443. doi:10.1038/s41586-020-2308-7 32461654 PMC7334197

[14] Richards S , Aziz N , Bale S , et al. Standards and guidelines for the interpretation of sequence variants: a joint consensus recommendation of the American College of Medical Genetics and Genomics and the Association for Molecular Pathology. Genet Med. 2015;17(5):405‐424. doi:10.1038/gim.2015.30 25741868 PMC4544753

[15] Biesecker LG , Harrison SM . ClinGen sequence variant interpretation working group. The ACMG/AMP reputable source criteria for the interpretation of sequence variants. Genet Med. 2018;20(12):1687‐1688. doi:10.1038/gim.2018.42 29543229 PMC6709533

[16] Lee‐Vaupel M , Conzelmann E . A simple chromogenic assay for arylsulfatase A. Clin Chim Acta. 1987;164(2):171‐180. doi:10.1016/0009-8981(87)90068-4 2885112

[17] Hiatt JB , Pritchard CC , Salipante SJ , O'Roak BJ , Shendure J . Single molecule molecular inversion probes for targeted, high‐accuracy detection of low‐frequency variation. Genome Res. 2013;23(5):843‐854. doi:10.1101/gr.147686.112 23382536 PMC3638140

[18] Sheth J , Mistri M , Sheth F , et al. Burden of lysosomal storage disorders in India: experience of 387 affected children from a single diagnostic facility. JIMD Rep. 2014;12:51‐63. doi:10.1007/8904_2013_244 23852624 PMC3897787

[19] Abstracts. J Inherit Metab Dis. 2022;45(S1):1‐870. doi:10.1002/jimd.12536 34855207

[20] Al‐Hassnan ZN , Al Dhalaan H , Patay Z , et al. Sphingolipid activator protein B deficiency: report of 9 Saudi patients and review of the literature. J Child Neurol. 2009;24(12):1513‐1519. doi:10.1177/0883073809341269 19955343

[21] Regis S , Filocamo M , Corsolini F , et al. An Asn > Lys substitution in saposin B involving a conserved amino acidic residue and leading to the loss of the single N‐glycosylation site in a patient with metachromatic leukodystrophy and normal arylsulphatase A activity. Eur J Hum Genet EJHG. 1999;7(2):125‐130. doi:10.1038/sj.ejhg.5200266 10196694

[22] Ittisoponpisan S , Islam SA , Khanna T , Alhuzimi E , David A , Sternberg MJE . Can predicted protein 3D structures provide reliable insights into whether missense variants are disease associated? J Mol Biol. 2019;431(11):2197‐2212. doi:10.1016/j.jmb.2019.04.009 30995449 PMC6544567

[23] Henseler M , Klein A , Reber M , Vanier MT , Landrieu P , Sandhoff K . Analysis of a splice‐site mutation in the sap‐precursor gene of a patient with metachromatic leukodystrophy. Am J Hum Genet. 1996;58(1):65‐74. 8554069 PMC1914953

[24] Grossi S , Regis S , Rosano C , et al. Molecular analysis of ARSA and PSAP genes in twenty‐one Italian patients with metachromatic leukodystrophy: identification and functional characterization of 11 novel ARSA alleles. Hum Mutat. 2008;29(11):E220‐E230. doi:10.1002/humu.20851 18693274

[25] Kuchar L , Ledvinová J , Hrebícek M , et al. Prosaposin deficiency and saposin B deficiency (activator‐deficient metachromatic leukodystrophy): report on two patients detected by analysis of urinary sphingolipids and carrying novel PSAP gene mutations. Am J med Genet A. 2009;149A(4):613‐621. doi:10.1002/ajmg.a.32712 19267410 PMC3437469

[26] Deconinck N , Messaaoui A , Ziereisen F , et al. Metachromatic leukodystrophy without arylsulfatase A deficiency: a new case of saposin‐B deficiency. Eur J Paediatr Neurol. 2008;12(1):46‐50. doi:10.1016/j.ejpn.2007.05.004 17616409

[27] Kretz KA , Carson GS , Morimoto S , Kishimoto Y , Fluharty AL , O'Brien JS . Characterization of a mutation in a family with saposin B deficiency: a glycosylation site defect. Proc Natl Acad Sci U S A. 1990;87(7):2541‐2544. doi:10.1073/pnas.87.7.2541 2320574 PMC53725

[28] Shapiro LJ , Aleck KA , Kaback MM , et al. Metachromatic leukodystrophy without arylsulfatase A deficiency. Pediatr Res. 1979;13(10):1179‐1181. doi:10.1203/00006450-197910000-00021 41211

[29] Holtschmidt H , Sandhoff K , Kwon HY , et al. Alternative splicing that generates three mRNAs and a newly found mutation responsible for a clinical disease. J Biol Chem. 1991;266(12):7556‐7560. 2019586

[30] Hahn AF , Gordon BA , Hinton GG , Gilbert JJ . A variant form of metachromatic leukodystrophy without arylsulfatase deficiency. Ann Neurol. 1982;12(1):33‐36. doi:10.1002/ana.410120106 6126151

[31] Zhang XL , Rafi MA , DeGala G , Wenger DA . Insertion in the mRNA of a metachromatic leukodystrophy patient with sphingolipid activator protein‐1 deficiency. Proc Natl Acad Sci U S A. 1990;87(4):1426‐1430. doi:10.1073/pnas.87.4.1426 1689485 PMC53488

[32] Zhang XL , Rafi MA , DeGala G , Wenger DA . The mechanism for a 33‐nucleotide insertion in mRNA causing sphingolipid activator protein (SAP‐1)‐deficient metachromatic leukodystrophy. Hum Genet. 1991;87(2):211‐215. doi:10.1007/BF00204185 2066109

[33] Siri L , Rossi A , Lanza F , et al. A novel homozygous splicing mutation in PSAP gene causes metachromatic leukodystrophy in two Moroccan brothers. Neurogenetics. 2014;15(2):101‐106. doi:10.1007/s10048-014-0390-4 24478108

