# Supplementary material for: Late infantile and adult‐onset metachromatic leukodystrophy due to novel missense variants in the PSAP gene: Case report from India
Source: JIMD Rep. 2023 Jun 5;64(4):265–73. doi: 10.1002/jmd2.12374 (PMC10315378; doi:10.1002/jmd2.12374)
Supplement: Supplementary file 1 — Data S1. Supporting Information [file JMD2-64-265-s001.docx]

**Supplementary Information**

**Supplementary Materials and Methods**

**Targeted exome sequencing methodology**

DNA from the proband was sent for targeted exome sequencing study. Target enrichment was performed using a custom capture kit targeting coding regions of approximately 4000 genes; the libraries were sequenced to mean > 80-100X coverage on the Illumina HiSeq sequencing platform (Illumina, USA). BWA was used to align the reads to the human reference genome assembly (GRCh37/hg19) and germline variants were called using GATK v3.6.^1,2^ Variant annotation was performed using VEP against the Ensemble release 87 human gene model.^3^

**Single molecule molecular inversion probes (smMIPs) based NGS study**

Target genomic regions were captured in a reaction containing smMIPs and genomic DNA in a molecular ratio of 1000:1. The resulting library was then sequenced on the Illumina MiSeq platform at a mean coverage of 200X. The obtained reads were aligned to the human reference genome assembly (GRCh37/hg19) using BWA and germline variants were called using GATK v3.6.^1,2^ Variants were annotated, filtered, and prioritized based on the proband’s phenotype (in HPO format) using Exomiser v12 ^4^ integrating data from SIFT ^5^, Polyphen2 ^6^, and MutationTaster ^7^, Combined Annotation Dependent Depletion (CADD) scores, dbSNP (www.ncbi.nlm.nih.gov/SNP/), the Genome Aggregation Database (gnomAD; gnomad.broadinstitute.org) and ClinVar ([www.ncbi.nlm.nih.gov/clinvar](http://www.ncbi.nlm.nih.gov/clinvar)).

**PCR and Sanger sequencing**

Variants identified in the *PSAP* gene were validated by PCR followed by Sanger sequencing in the parents of proband 1 and in proband 2. Primers were designed using Primer-3.^8^ For NM_002778.4, *PSAP* exon 6, the forward primer 5’ TCAGCAAGTGCCCCCTAAT 3’ and the reverse primer 5’ TTGTCTGAACGCCCTACTCC 3’ generated a 326 bp product encompassing the mutation. Amplification included 5-min denaturation at 94 °C followed by 30 cycles each consisting of 1 min denaturation at 94 °C for 45 s of annealing at temperature 62 °C, and 45 sec extension at 72 °C. Final extension was carried out at 72 °C for 10 min. PCR products were electrophoresed on 2% agarose with 100 base-pair DNA ladder. Products that passed this quality check were purified by treatment with Exo-SAP-IT™ (USB Corporation, OH, USA) and then sequenced using BigDye Terminator v3.1 cycle sequencing kit and capillary electrophoresis was performed using an automated sequencer SeqStudio (Applied Biosystems SeqStudio Genetic Analyzer).

**Supplementary Figures**

**Supplementary Figure 1**: Sanger sequencing chromatogram of the parents (1a: Mother, 1b: Father) of case 1 denoting heterozygous variant c.688T>G (pCys230Gly) shown by red arrow.


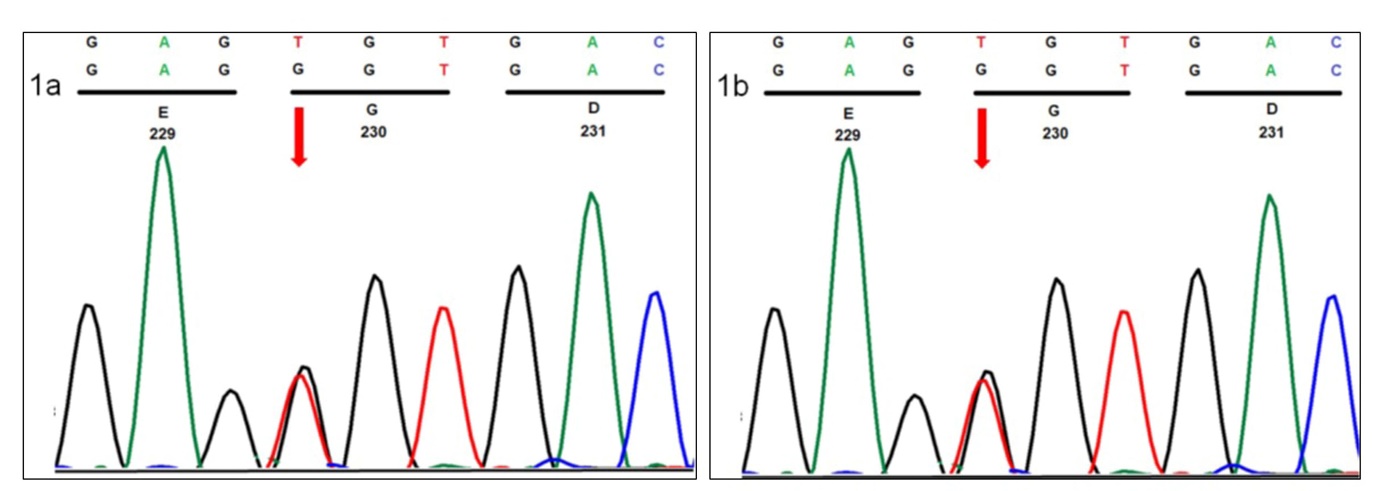


**Supplementary Figure 2**: Integrative Genomics Viewer (IGV) snapshot showing presence of the homozygous variant chr10-73587898-C-T in the proband (in red)


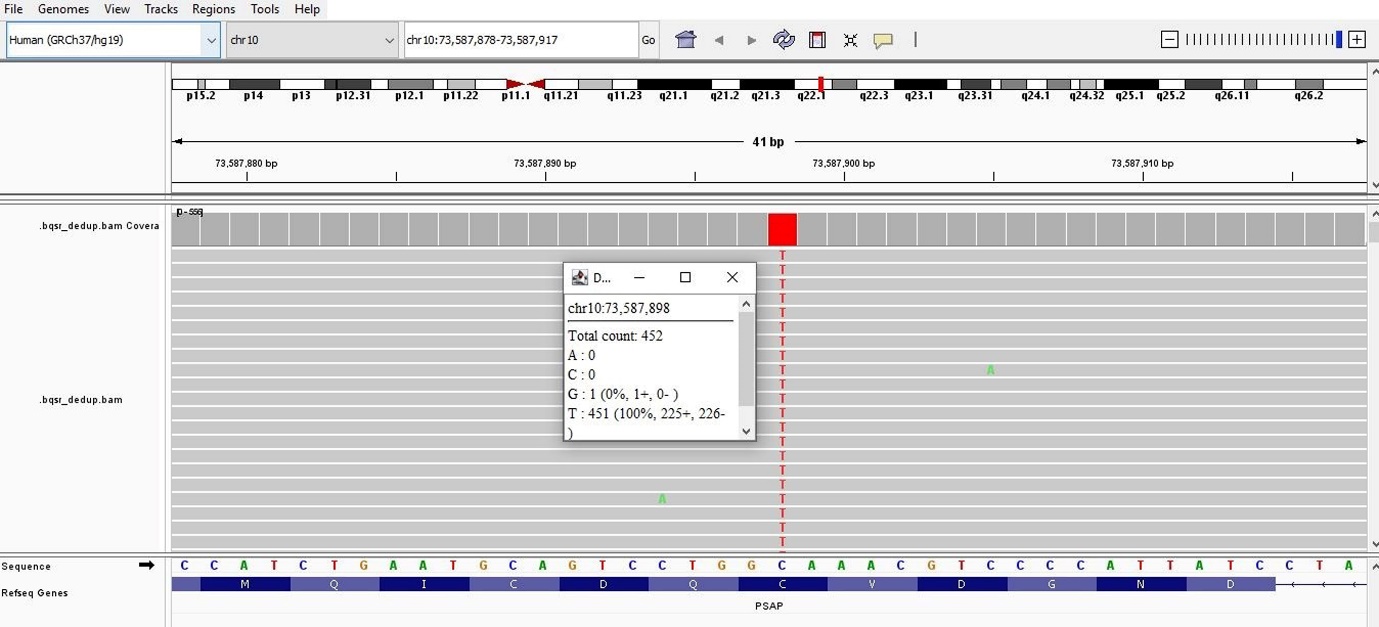


**Supplementary Figure 3**: Sanger sequencing chromatogram of the proband, red arrow denotes the variant c.593G>A (Cys198Tyr)


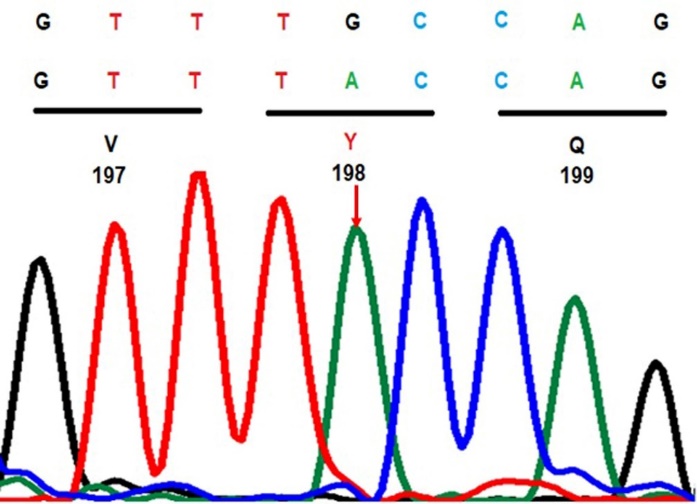


**References**

1. Li H, Durbin R. Fast and accurate short read alignment with Burrows-Wheeler transform. *Bioinforma Oxf Engl*. 2009;25(14):1754-1760. doi:10.1093/bioinformatics/btp324

2. McKenna A, Hanna M, Banks E, et al. The Genome Analysis Toolkit: a MapReduce framework for analyzing next-generation DNA sequencing data. *Genome Res*. 2010;20(9):1297-1303. doi:10.1101/gr.107524.110

3. McLaren W, Gil L, Hunt SE, et al. The Ensembl Variant Effect Predictor. *Genome Biol*. 2016;17(1):122. doi:10.1186/s13059-016-0974-4

4. Smedley D, Jacobsen JOB, Jäger M, et al. Next-generation diagnostics and disease-gene discovery with the Exomiser. *Nat Protoc*. 2015;10(12):2004-2015. doi:10.1038/nprot.2015.124

5. Kumar P, Henikoff S, Ng PC. Predicting the effects of coding non-synonymous variants on protein function using the SIFT algorithm. *Nat Protoc*. 2009;4(7):1073-1081. doi:10.1038/nprot.2009.86

6. Adzhubei IA, Schmidt S, Peshkin L, et al. A method and server for predicting damaging missense mutations. *Nat Methods*. 2010;7(4):248-249. doi:10.1038/nmeth0410-248

7. Schwarz JM, Rödelsperger C, Schuelke M, Seelow D. MutationTaster evaluates disease-causing potential of sequence alterations. *Nat Methods*. 2010;7(8):575-576. doi:10.1038/nmeth0810-575

8. Untergasser A, Cutcutache I, Koressaar T, et al. Primer3--new capabilities and interfaces. *Nucleic Acids Res*. 2012;40(15):e115. doi:10.1093/nar/gks596
